# Inter-Observer Reproducibility of [18F]FDG PET/CT Radiomic Features in Primary Breast Carcinoma

**DOI:** 10.3390/jimaging12070300

**Published:** 2026-07-04

**Authors:** Alexandru Mitoi, Raluca Mititelu, Cosmin Medar, Vlad Octavian Bolocan, Constantin Ciprian, Ioan-Nicolae Mateș

**Affiliations:** 1 Doctoral Program Studies, University of Medicine and Pharmacy “Carol Davila”, 050474 Bucharest, Romania; alexandru.mitoi@drd.umfcd.ro; 2 Department of Nuclear Medicine, Carol Davila University of Medicine and Pharmacy, 010825 Bucharest, Romania; raluca.mititelu@umfcd.ro; 3 Clinic of Nuclear Medicine, University Emergency Central Military Hospital “Dr. Carol Davila”, 010825 Bucharest, Romania; 4 Department of Fundamental Sciences, Faculty of Midwifery and Nursing, University of Medicine and Pharmacy “Carol Davila”, 050474 Bucharest, Romania; 5 Clinical Laboratory of Radiology and Medical Imaging, Clinical Hospital “Prof. Dr. Theodor Burghele”, 050664 Bucharest, Romania; 6 Department of Diabetes and Nutrition, Medicine Faculty, Titu Maiorescu University, 031593 Bucharest, Romania; ciprian_constantin@yahoo.com; 7 General and Esophageal Surgery Clinic, “Sfanta Maria” Clinical Hospital, Carol Davila University of Medicine and Pharmacy, 020021 Bucharest, Romania; ioan.mates@umfcd.ro

**Keywords:** PET/CT, [18F]FDG, breast carcinoma, radiomics, intraclass correlation coefficient, inter-observer reproducibility, segmentation

## Abstract

Radiomic feature stability is a necessary condition for clinical translation, yet the impact of inter-observer segmentation variability remains insufficiently characterized for [18F]FDG PET/CT in breast carcinoma. We evaluated the inter-observer reproducibility of 107 original radiomic features extracted from [18F]FDG PET/CT images of 42 patients with biopsy-proven, treatment-naive primary breast carcinoma, using an IBSI-aligned PyRadiomics workflow. Two nuclear medicine physicians independently segmented each tumor using semi-automatic Otsu thresholding to generate an initial tumor mask, followed by manual correction. Reproducibility was quantified using ICC(A,1) with bootstrap-derived 95% confidence intervals. A two-stage reproducibility and redundancy-based feature reduction strategy, combining an ICC threshold with Spearman correlation-based redundancy removal, was applied across nine threshold combinations, and features were classified into three pre-specified stability categories. The segmentation agreement was good, with a mean Dice coefficient of 0.847. Most features showed excellent reproducibility (81/107, 75.7% with ICC ≥ 0.90; median ICC 0.972), whereas shape features based on maximum lesion extension showed poor reproducibility (ICC 0.10–0.25). The reduction strategy resulted in 19 stable non-redundant features, with eight retained across all threshold combinations; 79 features (73.8%) met high-stability criteria. These results and the proposed stability classification framework provide a methodological basis for future predictive PET radiomics studies in breast carcinoma.

## 1. Introduction

### 1.1. Clinical Context

Breast cancer remains a major challenge for global public health due to its complex formation mechanisms, varied clinical manifestations, and multifactorial etiology, which involves genetic, environmental, and lifestyle factors [1]. The incidence of the disease is increasing globally, and breast cancer is responsible for approximately one-third of all neoplasms diagnosed in women, with a mortality rate of about 15% of all cases [2].

Invasive breast cancer encompasses a wide spectrum of histological subtypes, the most common being invasive breast carcinoma of no special type (NST), previously known as invasive ductal carcinoma, accounting for approximately 40–80% of cases [3]. The second most frequent subtype is invasive lobular carcinoma, alongside other forms with specific histological features, such as mucinous adenocarcinoma and invasive micropapillary carcinoma. Considering their prognostic and predictive importance, national and international guidelines recommend the evaluation of the biomarkers ER (estrogen receptor), PR (progesterone receptor), HER2 (human epidermal growth factor receptor 2), and Ki-67 [4,5,6]. In routine diagnostic practice, ER, PR, and Ki-67 are analyzed through immunohistochemistry, and HER2 status through immunohistochemistry and/or in situ hybridization [7,8].

Depending on the expression of hormone receptors (HR), HER2 status, and Ki-67 proliferation index, breast carcinoma can be classified into five molecular subtypes, which are frequently used in clinical practice. Approximately 70% of breast neoplasms express the estrogen receptor, and the majority of ER-positive tumors also express the progesterone receptor. ER-positive tumors are usually included in the luminal cancer category, which is further subdivided based on HER2 status and Ki-67 level [9,10,11,12].

Recently, PET/CT imaging with [18F]FDG has strengthened its role in the initial staging of locally advanced forms and in detecting recurrences [10,13].

The uptake of the radiopharmaceutical [18F]FDG varies depending on the histological and molecular characteristics of the tumor. This is lower in low- or intermediate-grade tumors (G1–G2), in tumors with low proliferation index (Low Ki-67), in tumors with positive hormone receptors, in invasive lobular carcinoma, in situ ductal carcinoma, and in the luminal A subtype. In contrast, uptake is higher in high-grade tumors (G3), with a high proliferation index (High Ki-67) and negative hormone receptors, in triple-negative tumors, in the luminal B subtype, and in invasive ductal carcinomas [12]. It is also well-known that [18F]FDG is not specific to malignant lesions; as increased uptake may also be seen in inflammatory processes, infections, or benign tumors, which reduces the specificity of the examination [14,15].

The metabolic parameters conventionally used in PET/CT imaging, SUVmax (maximum standardized uptake value), TLG (total lesion glycolysis), and MTV (metabolic tumor volume), have shown moderate correlations in some studies with immunohistochemical prognostic factors and tumor grade [16,17], highlighting the need for more effective imaging markers.

### 1.2. Radiomics in Breast Carcinoma [18F]FDG PET/CT

As an advanced method of medical image analysis, radiomics has the ability to transform digital images into quantitative data through the automatic extraction of a large number of imaging features, with the aim of quantifying the tumor phenotype and highlighting relevant information about the underlying pathophysiology [18,19]. For optimal results, an increasing number of authors recommend the use of a standardized workflow regarding image acquisition, reconstruction, segmentation, data extraction, and analysis [20,21].

Conventionally, radiomic features are grouped into three primary classes: shape, first-order (histogram statistics), and texture (co-occurrence, run-length, zone, dependence, and neighborhood grey level difference matrices); these features can quantify intratumoral metabolic heterogeneity and provide complementary information to conventional metabolic parameters [20,22].

In breast carcinoma, [18F]FDG PET/CT radiomics has been researched for characterizing the tumor biological profile, molecular subtypes, and estimating immunohistochemical biomarkers Ki-67 and HER2 [23,24,25,26].

Despite increasingly promising results, data from [18F]FDG PET/CT radiomics in breast carcinoma are still limited by methodological heterogeneity, small patient cohort sizes, and the lack of systematic external validation [27].

### 1.3. The Problem of Reproducibility and Standardization

The transition of radiomics into clinical practice is greatly limited by the high sensitivity of the features to fine variations that occur at any stage of the analysis process. Notable differences that affect the results are encountered with changes in the acquisition protocol, reconstruction method, voxel size, and grey level discretization [28,29].

Additionally, in PET/CT imaging, these sources of variability are complemented by sources of variability caused by the quantitative value of uptake. Changes also arise depending on the conversion of SUVs, the injected dose, the elapsed time post-radiopharmaceutical injection, the calibration of the scanner, the patient characteristics, and other factors.

The lower spatial resolution of PET compared to anatomical imaging methods can influence the delineation of lesions, especially in the case of small tumors or those with high uptake heterogeneity. In this context, the segmentation of the volume of interest and the evaluation of inter-observer reproducibility become a very important step in the radiomic workflow.

From the need for standardization of radiomic data reporting, Image Biomarker Standardization Initiative (IBSI) was conceptualized, which offers consensus definitions for 169 features and calibration values [30]. This initiative is complemented by the CheckList for EvaluAtion of Radiomics (CLEAR) guideline, which aims for transparent and complete reporting of radiomic studies [31], as well as broader initiatives for transparency and reproducibility in medical artificial intelligence [32].

### 1.4. Current State of Radiomic Reproducibility Research

The intraclass correlation coefficient (ICC) is the standard method for evaluating the reliability of radiomic features [33]. The study by Xue et al. of 47 radiomic studies analyzing the reliability of PET/CT exploration, however, highlighted a series of persistent methodological issues: ICC values are frequently reported partially, on small cohorts, without confidence intervals, and the selection criteria adopted vary significantly between studies (0.60 to 0.90), complicating the comparison of results and their transfer between clinical contexts [29].

Studies dedicated to radiomic reproducibility in breast cancer are predominantly focused on anatomical imaging, especially magnetic resonance imaging. In contrast, for [18F]FDG PET/CT, used for staging advanced loco-regional breast cancers as well as for evaluating treatment response, there is insufficient data on inter-observer reproducibility obtained on workflows that adhere to IBSI recommendations. Additionally, the systematic characterization of the stability of radiomic features across the seven major classes is incompletely documented in the literature [23,27].

### 1.5. Objectives

The present study aimed to:

1. Quantify the inter-observer reproducibility of original radiomic features extracted using a workflow aligned with IBSI principles from [18F]FDG PET/CT images of primary breast tumors, independently segmented by two nuclear medicine specialists.
2. Evaluate the results by applying successive criteria based on inter-observer stability (ICC) and redundancy (Spearman correlation) on the final set of candidate features.
3. Propose a predefined framework for classifying the radiomic feature stability into three levels, primarily based on the ICC values and their confidence intervals, with potential for transfer to other imaging modalities and tumor locations.

## 2. Materials and Methods

We conducted a single-center retrospective observational study at the “Dr. Carol Davila” Central Military Emergency University Hospital. The selected clinical examination period was between 1 January 2022 and 15 March 2026. The study protocol received approval from the Local Ethics Committee of the “Dr. Carol Davila” Central Military Emergency University Hospital (approval no. 763). The [18F]FDG PET/CT examinations were performed as part of routine clinical care during the period, and the retrospective analysis of anonymized data was carried out following this approval. The study was conducted in accordance with the Declaration of Helsinki [34]. Anonymized clinical and imaging data were used for research purposes in accordance with institutional regulations and the ethics committee approval. No part of this dataset has been used in any previous publication. The methodological reporting of the study followed the recommendations of the CLEAR guideline (the CLEAR Checklist is provided in the Supplementary Materials).

### 2.1. Patient Cohort

Following the application of the initial inclusion criteria, 52 consecutive patients diagnosed with primary breast carcinoma were selected. From the initial cohort, after verifying the integrity of the clinicopathological data, 42 patients met the necessary criteria to be included in the final statistics. More precisely, 10 patients were excluded from the final analysis due to missing histopathological and immunohistochemical data, which in some cases overlapped; for example, the Ki-67 proliferation index was unavailable for 9 patients, of which 6 patients had it as the only missing immunohistochemical parameter, and 3 patients had it missing alongside other features such as the hormonal receptor status and HER2 status data; additionally, the tumor histological grading was unavailable for 4 patients.

The initial inclusion criteria were as follows:

- Histopathological diagnosis of breast carcinoma confirmed by needle biopsy;
- Primary breast lesion visible on [18F]FDG PET/CT;
- Examination [18F]FDG PET/CT performed prior to any therapeutic intervention (surgical, systemic, or radiotherapeutic).

The final inclusion criterion was the availability of a complete set of clinicopathological data, including hormone receptor status (ER and PR), HER2 gene expression, Ki-67 proliferation index, and histological tumor grade.

The exclusion criteria were:

- Hormonal or chemotherapy treatment administered prior to the PET/CT examination;
- Prior breast surgery on the lesion of interest including excisional biopsy;
- Tumor not detectable by PET/CT imaging;
- Inadequate quality of PET/CT images for analysis.

Although ER/PR/HER2/Ki-67 data and histological grade were not directly necessary for evaluating inter-observer reproducibility, these variables were defined as inclusion criteria to ensure a uniformly characterized cohort. Reproducibility analyses were based solely on PET/CT imaging data and the two independent tumor segmentations. We acknowledge that this requirement may have influenced the final composition of the cohort, but the exclusions were related to the lack of clinicopathological or immunohistochemical data, rather than the quality of the PET/CT images, FDG avidity or lesion size.

**Figure 1:**
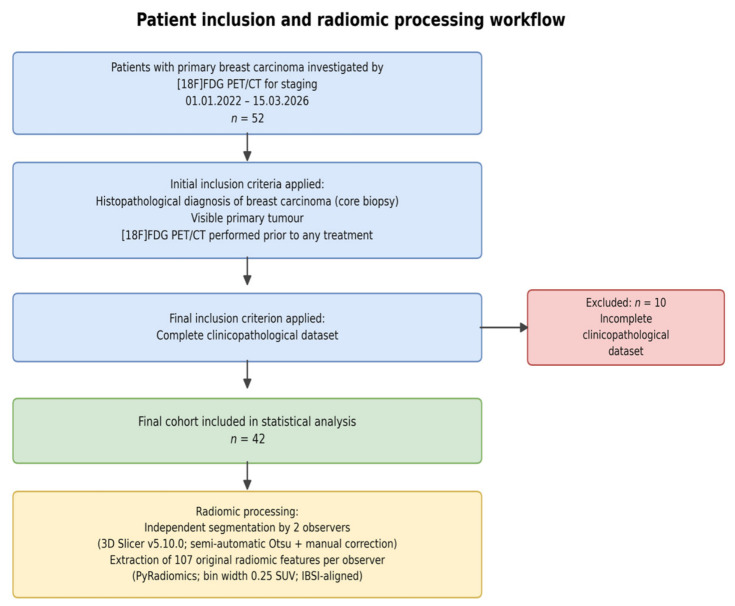
Flowchart for the inclusion of patients and radiomic processing.

The criteria for patient selection are presented in Figure 1.

### 2.2. [18F]FDG PET/CT Acquisition

All cases were investigated on the same General Electric (GE) Healthcare Discovery™ MI DR PET/CT system (GE HealthCare, Waukesha, WI, USA), based on a uniform clinical protocol, in accordance with the recommendations of the European Association of Nuclear Medicine (EANM) [35].

The patients were instructed to follow a low-carbohydrate diet for 24 h before the examination and to fast for at least 6 h. Capillary blood glucose was checked prior to the administration of the radiotracer (values < 200 mg/dL were accepted for inclusion). All patients received a dose of 2.5 MBq/kg of [18F]FDG. The dose was automatically calibrated (KARl100, Tema Sinergie, Faenza, Italy) and administered intravenously with an automatic syringe (RAD-INJECT) [36].

A waiting period of approximately one-hour post-injection followed, in a properly equipped room with a comfortable ambient temperature. The patients were instructed to remain calm during the waiting period and to hydrate with at least 1 L of water.

Prior to the investigation, the patients were asked to go to the restroom to empty their bladder, in order to reduce artefacts from physiological urinary uptake as well as to decrease the dose absorbed at the bladder level.

Image acquisition began at 60 ± 10 min, with the patient in a supine position and arms raised above the head, according to the vertex/tentorium to upper ⅓ of the thigh protocol. The PET reconstruction was performed using the OSEM (Ordered Subsets Expectation Maximisation) algorithm, GE VUE Point FX with SharpIR (VPFXS) with corrections for TOF (Time-of-Flight) and PSF (Point-Spread Function), matrix 256 × 256, and a native voxel 2.734 × 2.734 × 3.269 mm. The images were converted from activity units (Bq/mL) to standardized uptake values (SUV) normalized to body weight.

### 2.3. Tumor Segmentation

Tumor lesions were segmented as volume of interest (VOI) on PET image series. For precise localization of the uptake, the CT images performed for attenuation correction were also used as an anatomical reference. The PET images used were those based on mathematical attenuation correction (MAC), a choice motivated by the standardization of the process and the transferability of the workflow to future multicenter studies. The images were processed in the open-source application 3D Slicer v.5.10.0 (Surgical Planning Laboratory, Brigham and Women’s Hospital, Boston, MA, USA; open-source, https://www.slicer.org) [37,38].

Segmentation was performed using a semi-automated workflow in the Segment Editor module. First, Otsu [39] thresholding was applied to the whole PET volume using the “Threshold” effect with the automatic Otsu method and the “Threshold above” option. No observer-defined local bounding box or preselected local region of interest was used before applying Otsu thresholding. The resulting thresholded mask was used only as an initial candidate mask and was not considered the final tumor VOI.

The connected component corresponding to the primary breast lesion was then retained using the Islands effect with the “Keep selected island” option, while other disconnected areas of FDG uptake were removed. The mask was subsequently corrected manually using the Paint and Erase tools, with the attenuation-correction CT serving as the anatomical reference. Physiological or non-target uptake adjacent to the tumor, including myocardial, hepatic, skin, or pectoral muscle uptake, was excluded when it was contiguous with the candidate tumor mask. Regional lymph nodes were not included in the VOI, as the aim of the study was to analyze the primary breast lesion. Spatially separate nodal uptake was excluded by retaining only the island corresponding to the primary tumor, whereas nodal uptake contiguous or confluent with the primary lesion was removed manually along the anatomical boundary visible on CT.

A single VOI was analyzed for each patient. In cases of multifocal or multicentric disease, the dominant lesion, defined as the largest lesion and/or the lesion with the highest FDG uptake, was segmented. Confluent tumor foci without a clear separating boundary were segmented together as a single lesion complex. In the single bilateral case, the dominant lesion was selected for segmentation.

The two observers were a specialist and a senior physician, with a combined experience of over 20 years in interpreting oncological [18F]FDG PET/CT imaging. The same segmentation rules were applied independently by both observers. The observers did not have access to each other’s segmentations or to the histopathological and immunohistochemical results.

The complete segmentation and feature-extraction workflow in 3D Slicer is illustrated for a model case in Supplementary Materials (Figure S1).

### 2.4. Radiomic Feature Extraction

Radiomic features were extracted using the Radiomics extension in 3D Slicer (PyRadiomics v3.1.0 Computational Imaging and Bioinformatics Lab, Harvard Medical School/Brigham and Women’s Hospital, Boston, MA, USA; open-source) [40], following the IBSI recommendations for radiomics [30]. The discretization of grey levels was performed using the fixed bin width (FBW) method with a width of 0.25 SUV [41,42,43].

Because both gray-level discretization and voxel resampling may influence radiomic feature values, we evaluated the robustness of the inter-observer reproducibility estimates to these preprocessing choices. For this purpose, two sensitivity analyses were performed. First, features were re-extracted at three fixed bin widths (0.125, 0.25, and 0.5 SUV) without resampling. Second, as a confirmatory check of the native voxel anisotropy, features were re-extracted after isotropic resampling to 3 × 3 × 3 mm. The inter-observer ICC analysis was repeated for each configuration.

Isotropic resampling was not applied in the main analysis because all investigations were performed on the same PET/CT scanner with the same reconstruction protocol, resulting in a native voxel size of 2.734 × 2.734 × 3.269 mm with an anisotropy ratio of 1.195. In the case where investigations were conducted on multiple PET/CT machines, standardization of voxel sizes was necessary to limit the influence of spatial resolution variations on radiomic features [44].

A total of 107 radiomic features were extracted from the original images (without wavelet or Laplacian-of-Gaussian filters) and grouped into seven classes: 14 3D shape, 18 first-order, 24 Grey Level Co-occurrence Matrix (GLCM), 16 Grey Level Run Length Matrix (GLRLM), 16 Grey Level Size Zone Matrix (GLSZM), 14 Grey Level Dependence Matrix (GLDM), and 5 Neighbouring Grey Tone Difference Matrix (NGTDM). The complete list of extracted radiomic features is provided in Supplementary Materials Table S1. These correspond to the standardized IBSI protocols [30]. Each case was described with two parallel sets of 107 features, according to the independent segmentations of the two observers. Apart from the discretization (FBW of 0.25 SUV) and the absence of resampling and image filtering described above, all other PyRadiomics parameters were kept at their default configuration. The 3D Slicer workflow and the extraction-parameter table are provided in Supplementary Materials (Figure S1 and Table S2).

### 2.5. Reproducibility Analysis

The similarity of the two segmentations was verified using the Dice similarity coefficient (DSC) and the Jaccard index (JI) [29,45]. The numerical reproducibility of each radiomic feature was evaluated using the intraclass correlation coefficient ICC(A,1), two-way random effects, absolute agreement, and single rater [33]. The 95% confidence intervals for each ICC value were estimated using non-parametric bootstrap with 2000 iterations of resampling with replacement at the patient level. The interpretation of ICC values was based on the 95% confidence interval. ICC values below 0.50 were considered to have poor reproducibility, those between 0.50 and 0.75 with moderate reproducibility, those between 0.75 and 0.90 with good reproducibility, and values above 0.90 were considered to have excellent reproducibility [30,33].

For representative features from major radiomic classes, the Bland–Altman analysis was used to evaluate the absolute differences between observers [46].

### 2.6. Feature Selection, Sensitivity Analysis, and Stability Classification

A two-stage method for selecting relevant features was applied, following current methodological recommendations for radiomic studies [21,23].

- First stage—stability filter: Only features with an ICC ≥ 0.75 were carried forward to the redundancy-reduction step (Stage two). Features with lower inter-observer reproducibility (ICC < 0.75) were not propagated [33].
- Second stage—redundancy reduction: The 90 features retained after the stability filter were analyzed to reduce redundancy using a Spearman correlation matrix calculated between pairs of radiomic features based on the median value of the two observers for each feature. Pairs with |ρ| > 0.90 were considered highly redundant, and for each such pair, the feature with the higher ICC value was retained [9,21,23].

Redundancy reduction was performed with a deterministic, ICC-ranked greedy algorithm applied to the features that passed the stability filter (ICC ≥ 0.75). A pairwise Spearman correlation matrix was first computed across these features using, for each feature and patient, the per-observer average value (with two observers, this value is equivalent to the median). The absolute correlation coefficient, |ρ|, was used for redundancy assessment.

The surviving features were then sorted in descending order based on their ICC point estimate. Starting from the feature with the highest ICC, each not yet removed feature was retained, and all other not yet removed feature with |ρ| > 0.90 relative to that retained feature were removed as redundant. Once a feature had been removed, it was no longer allowed to remove other features. Thus, when a retained feature was directly correlated with several lower ICC features, it acted as the anchor and removed all of these directly redundant partners in the same pass.

The procedure is order-dependent by construction. However, the processing order is fully determined by the ICC ranking rather than by the input order of the features, making the outcome deterministic and reproducible. In the rare case of identical ICC values, ties were resolved by stable sorting, so no random tie-breaking was applied. Importantly, the per-observer average was used only to construct the Spearman correlation matrix. Feature ranking and retention decisions were based exclusively on the ICC values computed from the paired observer measurements.

The resulting set was defined as a set of stable non-redundant features, recommended for future clinical analyses. An additional supervised selection or predictive modeling step was not applied because the aim of the study is to evaluate reproducibility, not to develop a clinical model.

The robustness of the filtering method was evaluated by repeating the entire pipeline using alternative thresholds for ICC (0.75, 0.80 and 0.90) and for Spearman |ρ| (0.85, 0.90 and 0.95). The stability of the selection was quantified by the frequency with which each feature was retained across the 9 threshold combinations.

To provide an interpretable summary of radiomic feature stability, all 107 features were classified into three levels of stability by integrating both the ICC value and the estimated statistical uncertainty:

- High-stability features: excellent reproducibility, defined as ICC ≥ 0.90 and lower limit of the 95% CI > 0.75;
- Acceptable stability features: ICC ≥ 0.75, but not meeting the criteria for high-stability features;
- Unstable features ICC < 0.75.

These three categories represent a descriptive reproducibility summary applied to all 107 features and are independent of the feature-reduction workflow described above. Features classified as unstable (ICC < 0.75) are reported for completeness but were not propagated to the redundancy-reduction step.

### 2.7. Statistical Analysis

The statistical analysis was performed in Python (Python Software Foundation, Beaverton, OR, USA) 3.14 (using the NumPy, pandas, SciPy, statsmodels, and pingouin packages). Continuous variables were presented as mean ± standard deviation or as median with interquartile range, as appropriate; categorical variables were presented as absolute frequencies and percentages.

Comparisons between groups were performed using non-parametric tests (Kruskal–Wallis). Correlations were evaluated using the Spearman coefficient.

Inter-observer analysis was performed on all 42 cases, thus exceeding the guideline recommendation of including at least 30 heterogeneous cases for reproducibility studies evaluated by ICC [33]. Since the precision of the ICC estimate depends on the sample size, the results were reported along with 95% confidence intervals [47].

Statistical tests were performed bilaterally, without assuming a predetermined direction of the effect, with a statistical significance threshold set at α = 0.05.

## 3. Results

### 3.1. Characteristics of the Final Cohort

The final cohort included 42 patients with untreated primary breast carcinoma (Table 1). The median age was 68 years (IQR 50.5–73.5; range 36–84). The distribution by location was balanced: 21 patients with the tumor lesion in the right breast, 20 in the left breast, and 1 bilateral case. The predominant topographic location was in the supero-external quadrant (54.7%). The predominant histopathological type was invasive NST carcinoma, present in 37 patients (88.0%); the other 5 cases included invasive lobular carcinoma, mucinous carcinoma, encapsulated papillary carcinoma, and adenoid cystic carcinoma. The distribution of tumor grades was: G1—5 (11.9%), G2—27 (64.2%), G3—10 (23.8%). The hormonal receptor status was positive in 36 patients (85.7%), and HER2 was positive in 8 patients (19.0%). The Ki-67 proliferation index showed a wide distribution with a median of 35% (IQR 15–57.5%; range 2–80%).

The maximum tumor diameter, measured clinically by imaging, had a median of 25 mm (IQR 16–38; range 8–100 mm). The tumor volume quantified from PET segmentation had a median of 10.629 cm^3^ (IQR 5.111–23.287 cm^3^ with a range between 0.793 cm^3^ and 220.073 cm^3^). Segmented tumor volume was obtained from the PyRadiomics VoxelVolume feature, which accounts for native voxel dimensions by multiplying the number of voxels in the VOI by the physical voxel volume. The number of voxels per tumor VOI, calculated as the average of the two independent segmentations, ranged from 32 to 8959, with a median of 437 voxels. Six patients (14.3%) had breast lesions with an average tumor VOI of less than 100 voxels, calculated based on the two independent segmentations.

### 3.2. Analysis of Inter-Observer Segmentation Similarity

The two independent segmentations showed a high degree of similarity. The mean Dice coefficient was 0.847 ± 0.097 (median 0.857; IQR 0.795–0.922; range 0.607–0.987), and the mean Jaccard index was 0.747 ± 0.142 (median 0.750; IQR 0.660–0.856; range 0.435–0.976). Following the analyses, a high degree of similarity was observed in 31 out of the 42 (73.8%) patients according to the 0.80 threshold [29], 6 cases were between 0.70–0.80, and 5 cases were below 0.70, all corresponding to small lesions with mean tumor volumes under 5.25 cm^3^. No case showed a Dice score below 0.50.

**Figure 2:**
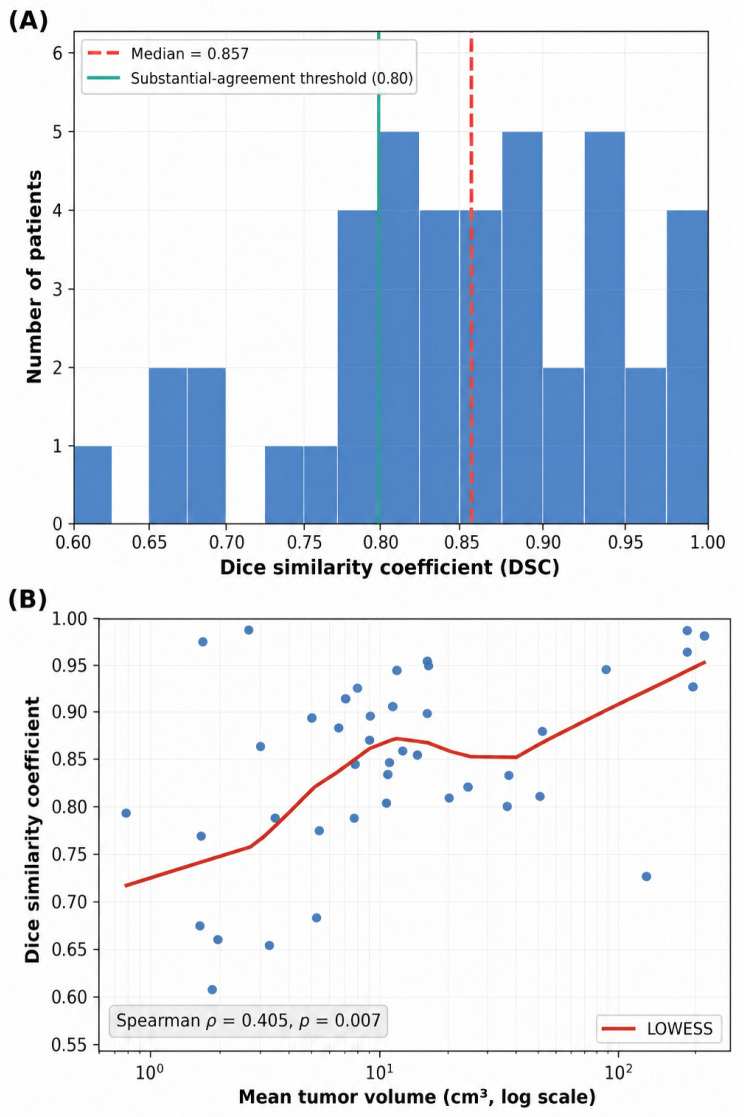
(**A**). The distribution of the Dice similarity coefficient across the 42 patients, with the median indicated (red line) and the standard threshold for substantial agreement 0.80 (green line). (**B**)**.** The relationship between the Dice similarity coefficient and mean tumor volume (cm^3^, logarithmic scale), showing a significant positive correlation (Spearman coefficient ρ = 0.405, *p* = 0.007). A non-parametric LOWESS smoothing curve is overlaid.

The inter-observer bias on tumor volume was not statistically significant, mean tumor volume for observer 1 was 35.882 cm^3^ and 33.201 cm^3^ for observer 2, with a mean difference of 2.680 cm^3^ (Wilcoxon test *p* = 0.785). The distribution of DSC per patient is represented in Figure 2A. A significant positive relationship was identified between DSC and tumor volume (Spearman ρ = 0.405, *p* = 0.007; Figure 2B), a pattern recognized in oncological imaging literature [28] and explained by the disproportionate impact of minor contour variations on DSC in small tumors. Stratified by volume categories, the mean DSC was 0.777 for lesions with volume < 5 cm^3^ (*N* = 10 cases), 0.858 for lesions between 5–50 cm^3^ (*N* = 24 cases) and 0.902 for lesions > 50 cm^3^ (*N* = 8 cases). This pattern suggested higher segmentation agreement in larger lesions, although variability remained present within each volume category.

### 3.3. Reproducibility of Radiomic Features

The ICC(A,1) values with 95% bootstrap confidence intervals (2000 iterations) were calculated for all 107 original radiomic features. The overall distribution across the Koo & Li [33] reliability categories was favorable for reproducibility: excellent (ICC ≥ 0.90)—81 features (75.7%); good (0.75–0.90)—9 (8.4%); moderate (0.50–0.75)—11 (10.3%); and poor (<0.50)—6 (5.6%) (Table 2). The median ICC value across all 107 features was 0.972 (IQR 0.904–0.991; range 0.100–0.999). Of the 81 features with excellent reproducibility, 79 (73.8% of all features) also had a lower 95% CI limit above 0.75 and were therefore classified as high-stability features.

**Figure 3:**
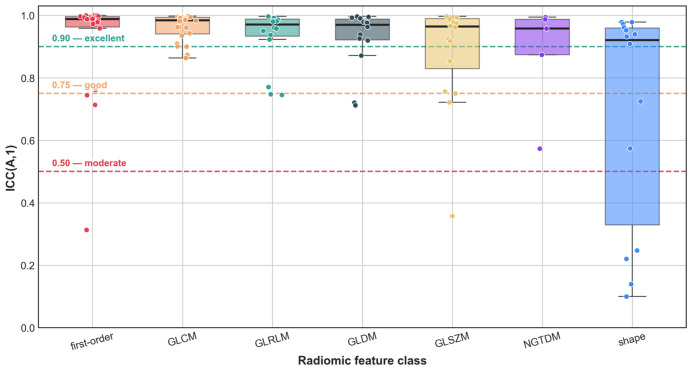
Distribution of ICC values stratified by the seven classes of radiomic features. The individual points (overlaid on the boxplots) represent the 107 features. The dashed horizontal lines indicate the interpretation thresholds of Koo & Li [33] (0.90—excellent; 0.75—good; 0.50—moderate). Inter-class variability is statistically significant (Kruskal–Wallis H = 15.638; *p* = 0.015). Notable is the substantial intra-class variability for the Shape class, contrasting with the more homogeneous distribution for first-order and GLCM.

Stratification across the seven feature classes (Table 3, Figure 3) revealed a pattern different from the initial classical hypothesis, with statistically significant inter-class variability (Kruskal–Wallis: H = 15.638, *p* = 0.015). First-order features (median ICC = 0.988) and GLCM (0.984) exhibited the highest reproducibility, followed by GLRLM (0.970), GLDM (0.969), GLSZM (0.964), and NGTDM (0.957). In contrast, the shape class exhibited the highest intra-class variability (median 0.920, but IQR 0.329–0.959—extremely wide), with 4 out of 14 features (28.6%) categorized as “poor” (ICC < 0.50).

The 4 shape features categorized as having poor reproducibility were all dependent on the maximum extension of the VOI or the principal axes of the shape (maximum diameter): Maximum2DDiameterRow (ICC = 0.100), Maximum3DDiameter (0.139), Maximum2DDiameterColumn (0.220), and MajorAxisLength (0.247). In contrast, within the same radiomic class, the features dependent on the entire voxel set of the VOI exhibited excellent reproducibility: VoxelVolume (ICC = 0.978), MeshVolume (0.978), SurfaceArea (0.961), and Sphericity (ICC = 0.908). This difference within the same class reflects the fact that the stability of the features in the Shape class depends on their mathematical definition, as described in Section 4.

**Figure 4:**
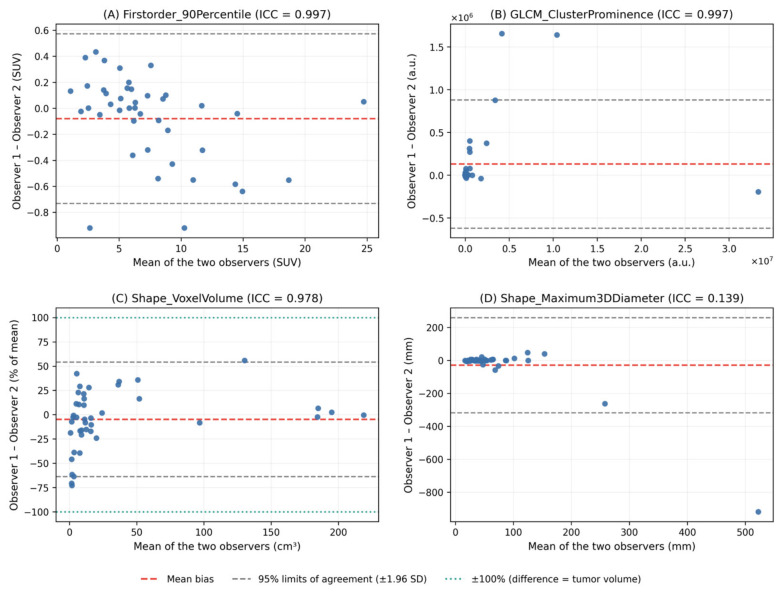
(**A**–**D**). Bland–Altman plots for four representative features. (**A**). Firstorder_90Percentile (ICC = 0.997, excellent reproducibility). (**B**). GLCM_ClusterProminence (ICC = 0.997, excellent reproducibility), (**C**). Shape_VoxelVolume (ICC = 0.978, excellent reproducibility), (**D**). Shape_Maximum3DDiameter (ICC = 0.139, poor reproducibility). The “x” axes show the mean value of the two observers, and the “y” axes show the observer1–observer2 difference, with feature-specific units indicated. In panel (**C**), VoxelVolume differences are expressed as percentage differences relative to the mean tumor volume, with dotted reference lines at ±100%. The dashed lines represent the mean bias (red) and the 95% agreement limits (gray, ±1.96 SD). The wider agreement limits for Maximum3DDiameter illustrate the sensitivity of this feature to minor variations in the tumor contour.

The Bland–Altman analysis conducted to compare the values of features extracted from the segmentations of the two observers (Figure 4) highlighted the absence of systematic inter-observer bias patterns for first-order and stable texture features, as well as the presence of very wide limits of agreement for maximum diameter shape features, in accordance with the observed ICC values.

### 3.4. Feature Selection

**Figure 5:**
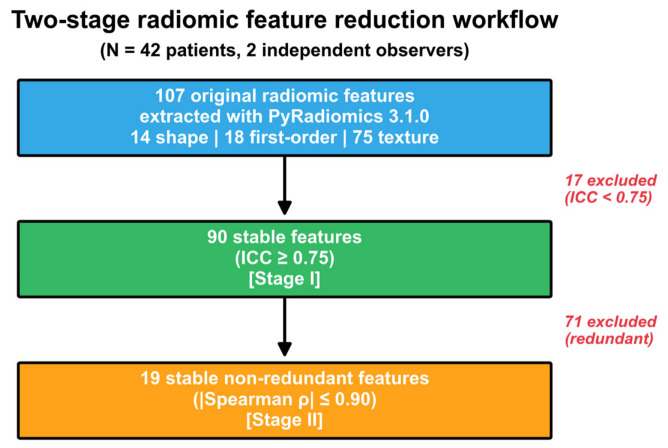
Flow diagram of radiomic feature filtering.

The application of successive feature selection progressively reduced the set of radiomic features (Figure 5).

Out of the 107 original features extracted with PyRadiomics, 90 met the stability criterion (ICC ≥ 0.75) and 19 were retained as a non-redundant stable candidate set (|Spearman ρ| ≤ 0.90).

In the first stage, inter-observer reproducibility was analyzed using ICC, and features with ICC ≥ 0.75 were considered stable. Following this analysis, 90 out of the initial 107 features (84.1%) were retained, thus eliminating 17 features with insufficient stability. The excluded features most frequently belonged to the shape class (6 features), followed by first-order and GLSZM (3 features each), GLDM and GLRLM (2 features each), and NGTDM (1 feature).

In the second stage, redundancy analysis based on Spearman correlation reduced the stable radiomic feature set from 90 to 19 non-redundant features by eliminating 71 features with redundant information. The final set included features from all seven radiomic classes: GLCM (5), GLDM (5), first-order (3), GLRLM (2), shape (2), NGTDM (1), and GLSZM (1) (Table 4).

The Spearman correlation matrix of the final set confirmed the absence of redundancy, with all pairwise correlation coefficients remaining within the predefined threshold (all values |ρ| ≤ 0.90; Figure S2 provided in Supplementary Materials).

### 3.5. Sensitivity Analyses on Thresholds

**Figure 6:**
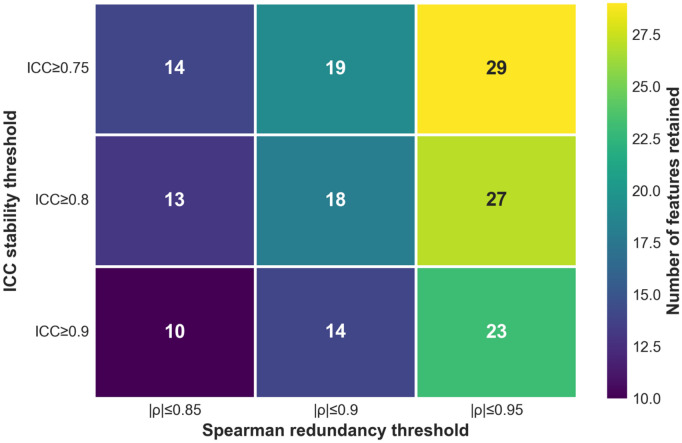
Heatmap of the number of features retained in the 9 combinations of ICC × |ρ| Spearman thresholds analyzed. The values in the cells represent the number of features after applying both sequential filters.

The stability of the filtering protocol was evaluated by repeating the entire procedure with the 9 threshold combinations (ICC × |ρ| Spearman). The number of retained features varied between 10 (the most restrictive combination: ICC ≥ 0.90 + |ρ| ≤ 0.85) and 29 (the most permissive: ICC ≥ 0.75 + |ρ| ≤ 0.95)—as shown in Figure 6.

A subset of 8 features was retained across all 9 threshold combinations, suggesting maximum robustness in the choice of specific thresholds (Table 5). These features represent the core of maximum stability of the radiomic pipeline in this cohort.

In addition to the threshold sensitivity analysis, we assessed the robustness of the reproducibility estimates to two preprocessing choices: gray-level discretization and voxel resampling. Across the three tested fixed bin widths (0.125, 0.25 and 0.5 SUV), the median ICC remained high (0.971, 0.972 and 0.962, respectively), and per-feature ICC values were strongly preserved compared with the reference configuration (Spearman ρ = 0.97–0.98). The reliability classification remained unchanged for 88–90% of features. The coarser bin width reduced the number of high-stability features (from 79 to 72), consistent with the greater sensitivity of small-volume lesions to discretization. After isotropic resampling to 3 × 3 × 3 mm, the median ICC remained essentially unchanged (0.976). The complete results are provided in Supplementary Table S3.

### 3.6. Identification of Highly Stable Features

Based on the prespecified criteria (ICC ≥ 0.90 and the lower limit of the 95% CI > 0.75), a subset of 79 highly stable features was identified. These exhibited ICC point values with excellent reproducibility, and the lower limit of the confidence interval was above the good reproducibility threshold. The subset of features represented 73.8% of the total 107 extracted features. The class distribution of stability categories was: GLCM 21/24 (87.5%), GLRLM 13/16 (81.2%), first-order 14/18 (77.8%), GLDM 10/14 (71.4%), GLSZM 10/16 (62.5%), NGTDM 3/5 (60.0%), and shape 8/14 (57.1%).

In the category of acceptable stability, 11 features (10.3%) were classified with ICC ≥ 0.75, but did not simultaneously meet the criteria for high stability.

In contrast, 17 features (15.9%) were considered unstable (ICC < 0.75) and are not recommended for use in future predictive models without further verification (Table 6). They predominantly included features dependent on extreme values or sensitive to changes in the lesion contour, especially related to the maximum extension of the VOI: Maximum2DDiameterRow/Column, Maximum3DDiameter, and MajorAxisLength.

The unstable set also included textural features emphasizing areas, dependencies, or runs with low gray levels coming from GLSZM, GLDM, GLRLM and NGTDM classes.

## 4. Discussion

Based on the results obtained, the study provides specific data on inter-observer reproducibility of radiomic features extracted through a method aligned with IBSI recommendations from a dedicated cohort of untreated breast cancer patients who underwent [18F]FDG PET/CT investigation.

The main results have been synthesized into four major observations.

- The global reproducibility of the radiomic features was favorable. Out of the 107 features, 81 (75.7%) exhibited excellent reproducibility (ICC ≥ 0.90). The spatial agreement between the two segmentations was consistent, with most cases achieving a good overlap of segmentations, with a mean Dice coefficient of 0.847. Based on high-stability criteria (ICC ≥ 0.90 and the lower limit of the 95% confidence interval > 0.75), 79 features (73.8%) were identified. The percentage of nearly 74% of features suggests that in our case, in a single-center study with standardized acquisition and reconstruction protocols, a considerable number of features can be robustly reproduced between two observers.
- The analysis of radiomic feature classes highlighted a pattern that differed from previous studies, in which shape features are generally more stable compared to texture features, which are sensitive to segmentation variations [28,29]. In our study, the radiomic features of intensity and texture had high median values (ICC ≥ 0.95), thus being the most stable (first-order, GLCM, GLRLM, GLDM, GLSZM, NGTDM). In contrast, the Shape class exhibited the highest intra-class variability.

More specifically, the shape features that integrate the information of the entire VOI, such as VoxelVolume, MeshVolume, SurfaceArea, and Sphericity, were stable, while those dependent on the maximum contour extension, such as Maximum2DDiameterRow, Maximum2DDiameterColumn, Maximum3DDiameter, and MajorAxisLength, exhibited reduced reproducibility.

This difference has a direct mathematical explanation: the maximum diameters and principal axes measurements are influenced by the position of some extreme points of the VOI [30].

Thus, the inclusion or exclusion of a small number of peripheral voxels can modify the feature value, even if the overall overlap between segmentations remains good.

The practical implication is that the selection of radiomic features should not be based on membership in a class considered “stable,” but rather on the assessment of the reproducibility of each individual feature. This result supports the use of a stability filter at the individual feature level and justifies our approach of classifying features based on ICC and estimation uncertainty.

- The application of sequential stage filtering allowed for the reduction of the number of features without introducing a supervised modeling stage. The interobserver stability filter reduced the initial 107 features to 90 stable features. Subsequently, using Spearman correlation a final set of 19 stable non-redundant features was identified. This final set had representatives from the seven studied radiomic classes, suggesting that redundancy reduction did not completely eliminate any category. Moreover, 8 features were selected in all 9 threshold combinations tested in the sensitivity analysis, resulting in a core of maximum stability for the proposed workflow.
- The 79 highly stable radiomic features and the final subset of 19 stable non-redundant features provide a methodological basis for subsequent radiomic studies. These should not be interpreted as validated imaging biomarkers, but rather as an exploratory set, that may be suitable for the development of predictive or associative models, provided that external validation is performed and the features are integrated with relevant clinicopathological data.

### 4.1. Comparison with Existing Literature

Our results are partially aligned with data from the specialized literature regarding radiomic reproducibility, but they also highlight specific particularities. The systematic review by Xue et al., which included 481 radiomic studies that evaluated ICC, of which only 47 were PET studies, highlighted a marked heterogeneity of results, influenced by imaging modality, lesion, sample size, and the radiomic classes studied [29]. Shape features are usually considered stable, but the results available so far do not allow for the generalization of stability at the class level without a specific evaluation at the individual characteristic level. In our study, this observation is partially confirmed. The shape class had a median ICC of 0.920, which places it in the category of excellent reproducibility. But the intra-class analysis showed significant variability among shape features, with subgroups exhibiting very clear differences between those analyzing information from the entire VOI and those that depend on the maximum contour extension.

For intensity features and texture classes, our results align with the literature, with median ICC values above 0.95 for first-order, GLCM, GLRLM, GLDM, GLSZM, and NGTDM. These values can be partially explained by the monocentric design of the study, the acquisition of images according to a single protocol on a single PET/CT scanner, as well as the use of a standardized segmentation procedure. Through these, we eliminated the main source of variability (multi-vendor, multi-protocol) that usually affects multi-center studies and allowed us to more directly evaluate the inter-observer component of variability.

The methodological choice of ICC thresholds ≥ 0.75 for defining reproducible features is consistently supported by the literature [33]. Reducing redundancy through Spearman correlation is also aligned with the methodological practice used in radiomic studies to limit collinearity and overfitting of the variable set [21,28].

Additionally, the sensitivity analysis on 9 combinations of ICC and Spearman thresholds reinforces the methodological robustness of the workflow. The identification of a core set of 8 features retained regardless of the specific threshold combinations adopted suggests that this subset does not depend solely on the arbitrary choice of thresholds but rather reflects a group of features with high stability in the studied context [28].

### 4.2. Implications for Standardized Radiomic Reporting

The IBSI [30] and the CLEAR guideline [31] have highlighted in recent years the need for standardization of both the definitions of radiomic features and the reporting of studies that utilize them. Considering that IBSI provides definitions and reference values for 169 radiomic features and CLEAR proposes the use of a structured reporting framework, the prespecified classification into 3 levels of stability used in our study represents a proposal for reporting reproducibility at the individual feature level. Through this classification, in addition to the point value of the ICC, we also integrated statistical uncertainty through the lower limit of the 95% confidence interval, which allowed for differentiation between features with stability and those with seemingly favorable ICC values but with uncertain estimates.

The adoption of such a prespecified classification in future radiomic studies could reduce practical variability in the selection of candidate features and facilitate the comparison of results between studies, contributing to overcoming one of the main limitations identified in the literature [29].

In this context, the 79 highly stable features identified in our study constitute a methodologically documented set for the construction of subsequent predictive models in PET/CT with [18F]FDG in breast cancer, whether for predicting Ki-67, HER2 status, molecular subtypes, or response to neoadjuvant chemotherapy [23,27].

### 4.3. Biological Considerations on Stable Features

A significant proportion of the highly stable features originated from the first-order and GLCM classes, which has both methodological and clinical implications.

First-order features describe the global distribution of SUVs within the tumor VOI, including features such as percentiles (90th Percentile), energy (Energy and TotalEnergy), variance, and distribution asymmetry (Skewness). These features do not provide spatial data about the tumor texture, but they can reflect metabolic activity and the distribution of metabolic uptake. Thus, in this context, we can interpret 90Percentile as a descriptor of metabolic uptake without it being equivalent to SUVpeak. The relationship between [18F]FDG uptake and tumor proliferation rate is supported by the literature on breast cancer, with moderate correlations that do not allow the use of conventional PET parameters as independent biomarkers of proliferation [16,20].

Stable GLCM features describe local spatial relationships between grey levels, specifically how voxels with similar or different intensities are distributed in the neighborhood according to the radiomic definitions proposed by IBSI (30). From a biological perspective, this set can be interpreted as descriptors of intra-tumoral metabolic heterogeneity on [18F]FDG PET/CT images.

In the literature dedicated to breast carcinoma, PET/CT metabolic radiomics have been associated with histopathological biomarkers, response to neoadjuvant chemotherapy, and recurrence risk, indicating that metabolic heterogeneity may contain information regarding biological characterization and tumor prognosis [48].

However, the biological interpretation of textural features remains indirect and requires validation through histopathological or molecular correlation [48].

In contrast, features with low reproducibility from the GLSZM, GLDM, GLRLM, and NGTDM classes, especially those that emphasize areas, dependencies, or runs with low grey levels, may be more sensitive to the inclusion or exclusion of peripheral regions with low uptake. This sensitivity is possible in the context of PET segmentation, as tumor margins often contain voxels with low uptake or are affected by partial volume artefacts, and small contour differences can alter the proportion of these regions in the VOI. Thus, the instability of these features should not necessarily be interpreted as a lack of biological relevance but rather as a methodological limitation that necessitates verifying reproducibility before using them in predictive models [49,50].

### 4.4. Strengths of the Study

Our study presents methodological features worth mentioning.

- Through the single-scanner, single-protocol study design, the inter-scanner variability faced by most multi-center radiomic studies is eliminated. This has allowed us to isolate interobserver variability.
- The extraction workflow is aligned with IBSI recommendations, and the methodological reporting meets CLEAR requirements.
- Both observers performed the segmentations independently for all 42 patients.
- The 95% confidence intervals were estimated using bootstrap for all 107 ICC values, surpassing the practice commonly found in the literature that frequently reports only point estimates.
- Sensitivity analyses at 9 threshold combinations explicitly led to the identification of the selected robust features.
- The classification into predefined stability levels (high, acceptable, unstable) provides a standardized reporting tool for stability, transferable to other radiomic studies.

### 4.5. Limitations

- The study presented several limitations that must be acknowledged for academic transparency.
- Being a single-center retrospective study focused on a specific population, generalizability is limited. There is a need for validation on external multicentric cohorts with different scanners and protocols.
- Although the cohort size (*n* = 42) is adequate for estimating ICC with reasonable precision [33,47], it limits the statistical power for stratified analyses across different subgroups (molecular subtype, volume category).
- Although the use of the semi-automatic Otsu threshold followed by manual correction is a frequently used approach in PET lesion segmentation, it does not represent a universal standard. Additionally, in our workflow, the Otsu threshold was applied to the entire PET volume and, therefore, it may be influenced by the physiological distribution of the tracer outside the tumor. However, this step was only used to generate an initial mask. The final VOI was obtained after selecting the islands specific to the lesions and manual correction guided by CT. Consequently, segmentations based on alternative methods, such as a fixed threshold of 40% SUVmax or gradient-based approaches, could generate different reproducibility patterns.
- Six patients (14.3%) presented breast lesions with an average tumor VOI of less than 2.4 cm^3^, calculated based on the two independent segmentations. Even though we did not identify a universally accepted threshold for the minimum size of the VOI in radiomic analysis with PyRadiomics, reduced tumor volumes can affect the stability of texture features. In other words, the textural features derived from these small-sized lesions should be interpreted with caution [51].
- The analysis was restricted to the “original” features (without wavelet or Laplacian-of-Gaussian filters) to maintain direct physical interpretability.
- Finally, the study focused exclusively on inter-observer variability. Intra-observer reproducibility, test–retest repeatability and robustness to segmentation variability represent complementary directions for future research.

### 4.6. Future Directions

Future directions include the external validation of the high-stability feature set. To evaluate the robustness of these features under various technical and clinical conditions, the use of larger multicentric cohorts and varied PET/CT protocols is necessary.

Additionally, the analysis could be extended to radiomic features derived from filtered images, including those based on Wavelet and Laplacian-of-Gaussian transformations, to determine whether these additional classes provide complementary information without compromising inter-observer reproducibility [40,52].

Another direction that can be pursued is the use of stable and non-redundant features in supervised predictive models, oriented towards relevant clinicopathological variables, molecular subtypes, HER2 status, Ki-67 index, or response to neoadjuvant chemotherapy treatment. Such models require sufficiently large cohorts, validated in multicenter settings, and reported according to the TRIPOD+AI (Transparent Reporting of a multivariable prediction model for Individual Prognosis Or Diagnosis + Artificial Intelligence) [53] recommendations, considering that this updated guideline is intended for reporting prediction models developed through regression or machine learning methods.

In addition, future studies should evaluate more standardized tumor segmentation methods, including semi-automated, interactive, or artificial intelligence-assisted approaches, with the aim of reducing inter-observer variability and standardizing the pipeline. Recently, interactive 3D segmentation solutions have been proposed as open-source methods for segmenting complex medical images, and U-Net-based convolutional network models have been applied in oncological imaging for segmentation and classification tasks, with performance evaluation through metrics such as the Dice coefficient [38,54].

The integration of artificial intelligence-assisted methods into the radiomic workflow does not eliminate the need for external validation, transparent reporting, and robustness evaluation under real-world conditions. These challenges of translating information obtained from radiomic analysis into current clinical practice have also been highlighted in other areas of oncological imaging, where the high performance reported in experimental contexts does not necessarily translate into clinical implementation [55].

Additionally, the results of the study can serve as a methodological reference for the subsequent comparison of the Otsu segmentation method against the fixed threshold of 40% of SUVmax [56], gradient-based methods, and deep learning-assisted segmentation to evaluate their impact on the reproducibility of radiomic features in [18F]FDG PET/CT of breast carcinoma.

## 5. Conclusions

1. Through the obtained results, our study provides specific data on inter-observer reproducibility of radiomic features extracted through a workflow developed according to IBSI recommendations from [18F]FDG PET/CT imaging of untreated primary breast carcinoma.
2. The overall reproducibility was favorable (75.7% of features with ICC ≥ 0.90, with a mean Dice index of 0.847). There is also an important finding regarding the intra-class variability of shape features: maximum diameter-type features (mathematically dependent on the position of extreme points of the tumor contour) exhibited poor reproducibility, while features dependent on the entire volume are among the most stable.
3. From the stability-redundancy filtering method, a set of 19 stable non-redundant radiomic features was identified, with a core of 8 robust features across all tested threshold combinations. The pre-specified classification into three levels of radiomic feature stability proposes a transferable framework for reporting radiomic feature stability.
4. The 79 high-stability features identified by our study provide a starting point for future predictive research in [18F]FDG PET/CT radiomics of breast carcinoma and for overcoming one of the main methodological limitations of the current literature: the use of insufficiently validated stability radiomic features.

## Figures and Tables

**Table 1:** Cohort characteristics.

Characteristic	Value
Age (years)	68 (50.5–73.5; 36–84)
Tumor laterality: right/left/bilateral	21/20/1
Upper outer quadrant	23 (54.7%)
Histological type: invasive carcinoma NST	37 (88.0%)
Tumor grade: G1/G2/G3	5/27/10
HR-positive	36 (85.7%)
HER2-positive	8 (19.0%)
Ki-67 (%)	35 (15–57.5; 2–80)
Ki-67 ≥ 30%	22 (52.3%)
Maximum tumor diameter (mm)	25 (16–38; 8–100)
Segmented tumor volume (cm^3^)	10.629 (5.111–23.287; 0.793–220.073)
Small lesions, mean VOI volume < 2 cm^3^	6 (14.3%)

Demographic, clinicopathological, and immunohistochemical characteristics of the cohort (*N* = 42). Continuous variables are reported as median (IQR; range), categorical variables as absolute frequencies (percentage).

**Table 2:** Distribution of the 107 radiomic features across reliability categories.

ICC Category	Interval	*N*	%
Excellent	≥0.90	81	75.7%
Good	0.75–0.90	9	8.4%
Moderate	0.50–0.75	11	10.3%
Poor	<0.50	6	5.6%
Total		107	100%

**Table 3:** ICC reproducibility by radiomic feature class.

Class	*N*	Median ICC	Q1–Q3	Excellent	Good	Moderate	Poor	High-Stability Features
First-order	18	0.988	0.962–0.995	14	1	2	1	14
GLCM	24	0.984	0.940–0.993	21	3	0	0	21
GLRLM	16	0.970	0.933–0.987	13	1	2	0	13
GLDM	14	0.969	0.921–0.987	11	1	2	0	10
GLSZM	16	0.964	0.829–0.989	11	2	2	1	10
NGTDM	5	0.957	0.873–0.987	3	1	1	0	3
Shape	14	0.920	0.329–0.959	8	0	2	4	8
Total	107	0.972	0.904–0.991	81	9	11	6	79

Statistical summary of ICC values stratified by the seven classes of radiomic features. The median and interquartile range (Q1–Q3) are reported alongside the number of features per reliability category. The ICC values and 95% confidence intervals are presented to three decimal places by truncation; the classification of features into stability categories was performed based on the complete, unrounded values.

**Table 4:** The final candidate set of 19 stable non-redundant radiomic features.

Feature	Class	ICC	95% CI	Stability Category
firstorder_TotalEnergy	first-order	0.999	[0.995; 0.999]	High stability
firstorder_90Percentile	first-order	0.997	[0.994; 0.998]	High stability
glcm_ClusterShade	GLCM	0.981	[0.769; 0.994]	High stability
glrlm_GrayLevelNonUniformity	GLRLM	0.980	[0.952; 0.997]	High stability
ngtdm_Strength	NGTDM	0.987	[0.959; 0.996]	High stability
shape_Maximum2DDiameterSlice	shape	0.932	[0.840; 0.983]	High stability
glcm_Imc1	GLCM	0.934	[0.872; 0.973]	High stability
shape_Sphericity	shape	0.908	[0.791; 0.965]	High stability
glcm_Idn	GLCM	0.909	[0.831; 0.951]	High stability
glcm_Correlation	GLCM	0.873	[0.794; 0.923]	Acceptable stability
gldm_DependenceVariance	GLDM	0.926	[0.661; 0.968]	Acceptable stability
gldm_SmallDependenceEmphasis	GLDM	0.979	[0.962; 0.988]	High stability
glszm_LowGrayLevelZoneEmphasis	GLSZM	0.853	[0.670; 0.930]	Acceptable stability
gldm_LargeDependenceLowGrayLevelEmphasis	GLDM	0.871	[0.515; 0.928]	Acceptable stability
gldm_LargeDependenceHighGrayLevelEmphasis	GLDM	0.976	[0.785; 0.994]	High stability
gldm_DependenceNonUniformityNormalized	GLDM	0.939	[0.871; 0.972]	High stability
glcm_MCC	GLCM	0.863	[0.713; 0.936]	Acceptable stability
glrlm_RunVariance	GLRLM	0.922	[0.786; 0.990]	High stability
firstorder_Skewness	first-order	0.761	[0.564; 0.896]	Acceptable stability

The final candidate set of 19 stable non-redundant radiomic features, with ICC values, 95% confidence intervals, and inter-observer stability category. The ICC values and 95% confidence intervals are presented to three decimal places by truncation; the classification of features into stability categories was performed based on the complete, unrounded values.

**Table 5:** The 8 radiomic features retained in all 9 threshold combinations of the sensitivity analysis (ICC ∈ {0.75; 0.80; 0.90} × |ρ Spearman| ∈ {0.85; 0.90; 0.95}). These features constitute the core of maximum stability recommended as a priority for future research.

Feature	Class	ICC	95% CI	Stability Category
firstorder_TotalEnergy	first-order	0.999	[0.995; 0.999]	High stability
ngtdm_Strength	NGTDM	0.987	[0.959; 0.996]	High stability
glcm_ClusterShade	GLCM	0.981	[0.769; 0.994]	High stability
glrlm_GrayLevelNonUniformity	GLRLM	0.980	[0.952; 0.997]	High stability
glcm_Imc1	GLCM	0.934	[0.872; 0.973]	High stability
gldm_DependenceVariance	GLDM	0.926	[0.661; 0.968]	Acceptable stability
glcm_Idn	GLCM	0.909	[0.831; 0.951]	High stability
shape_Sphericity	shape	0.908	[0.791; 0.965]	High stability

The ICC values and 95% confidence intervals are presented to three decimal places by truncation; the classification of features into stability categories was performed based on the complete, unrounded values.

**Table 6:** The 17 unstable features (ICC < 0.75).

Feature	Class	ICC	95% CI
shape_Maximum2DDiameterRow	Shape	0.100	[0.047; 0.925]
shape_Maximum3DDiameter	Shape	0.139	[0.050; 0.924]
shape_Maximum2DDiameterColumn	Shape	0.220	[0.089; 0.967]
shape_MajorAxisLength	Shape	0.247	[0.098; 0.947]
firstorder_Minimum	First-order	0.313	[0.077; 0.598]
glszm_LargeAreaLowGrayLevelEmphasis	GLSZM	0.357	[0.206; 0.813]
ngtdm_Busyness	NGTDM	0.573	[0.523; 0.792]
shape_Elongation	Shape	0.574	[0.261; 0.867]
gldm_SmallDependenceLowGrayLevelEmphasis	GLDM	0.711	[0.411; 0.860]
firstorder_10Percentile	First-order	0.713	[0.522; 0.844]
gldm_LowGrayLevelEmphasis	GLDM	0.720	[0.536; 0.817]
glszm_SmallAreaLowGrayLevelEmphasis	GLSZM	0.721	[0.484; 0.814]
shape_Flatness	Shape	0.724	[0.453; 0.930]
firstorder_Kurtosis	First-order	0.744	[0.516; 0.885]
glrlm_ShortRunLowGrayLevelEmphasis	GLRLM	0.745	[0.548; 0.844]
glrlm_LowGrayLevelRunEmphasis	GLRLM	0.748	[0.562; 0.835]
glszm_LargeAreaHighGrayLevelEmphasis	GLSZM	0.749	[0.487; 0.955]

The ICC values and 95% confidence intervals are presented to three decimal places by truncation; the classification of features into stability categories was performed based on the complete, unrounded values.

## Data Availability

The clinical and imaging datasets analyzed in this study are not publicly available due to patient privacy, ethical and institutional restrictions.

## Supplementary Materials

The following supporting information can be downloaded at https://www.mdpi.com/article/10.3390/jimaging12070300/s1. Table S1. Complete list of the 107 radiomic features; Table S2. PyRadiomics extraction parameters (image type, feature classes, bin width, resampling, normalization, mask handling); Table S3. Inter-observer reproducibility sensitivity analysis (bin width and isotropic resampling); Figure S1. Multi-panel 3D Slicer segmentation and radiomics workflow; Figure S2. Spearman correlation matrix of the final set of 19 stable non-redundant radiomic features. Correlations were calculated using the median value of the two observers for each feature. The final set was obtained using ICC ≥ 0.75 for the stability filter and |ρ| > 0.90 for redundancy removal; therefore, all retained pairwise correlations were ≤0.90 in absolute value. Displayed values are rounded to two decimals. CLEAR Checklist.

## Abbreviations

The following abbreviations are used in this manuscript:

Abbreviation	Definition
CI	Confidence interval
CLEAR	CheckList for EvaluAtion of Radiomics research
DSC	Dice similarity coefficient
EANM	European Association of Nuclear Medicine
ER	Estrogen receptor
FBW	Fixed bin width
[18F]FDG	2-deoxy-2-[^18^F]fluoro-D-glucose
FWHM	Full width at half maximum
GE	General Electric
GLCM	Grey level co-occurrence matrix
GLDM	Grey level dependence matrix
GLRLM	Grey level run length matrix
GLSZM	Grey level size zone matrix
HER2	Human epidermal growth factor receptor 2
HR	Hormone receptor
IBSI	Image Biomarker Standardisation Initiative
ICC	Intraclass correlation coefficient
IQR	Interquartile range
JI	Jaccard index
Ki-67	Marker of proliferation Ki-67
MAC	Mathematical attenuation correction
MBq/kg	Megabecquerel per kilogram
MTV	Metabolic tumour volume
NGTDM	Neighbouring grey tone difference matrix
NST	No special type
OSEM	Ordered subsets expectation maximisation
PET/CT	Positron emission tomography/computed tomography
PR	Progesterone receptor
PSF	Point-spread function
SUV	Standardized uptake value
SUVmax	Maximum standardized uptake value
TLG	Total lesion glycolysis
TOF	Time-of-flight
VOI	Volume of interest
VPFXS	GE VUE Point FX with SharpIR
