# Supplementary material for: Inter-Observer Reproducibility of [18F]FDG PET/CT Radiomic Features in Primary Breast Carcinoma
Source: J Imaging. 2026 Jul 4;12(7):300. doi: 10.3390/jimaging12070300 (PMC13413217; doi:10.3390/jimaging12070300)
Supplement: Supplementary file 1 [file jimaging-12-00300-s001.zip › Table S2.pdf]

Table S2. Radiomic feature extraction parameters (PyRadiomics).
Feature extraction was performed with PyRadiomics through the SlicerRadiomics extension in 3D Slicer 5.10.0. The configuration below corresponds to the primary analysis and is provided as a structured parameter table to improve reproducibility.

Parameter	Value	Notes
Software	PyRadiomics (3D Slicer 5.10.0, SlicerRadiomics)	IBSI- aligned feature definitions
Image type	Original only	No wavelet / no Laplacian-of-Gaussian filtering
Feature classes	shape, firstorder, glcm, glrlm, glszm, gldm, ngtdm	107 features in total
Discretization	Fixed bin width = 0.25 SUV	—
Spatial resampling	None<br>Native voxel 2.734 × 2.734 × 3.269 mm	See sensitivity / isotropic analyses (Table S3)
Intensity normalization	Disabled	PET data already expressed in SUV
Extraction mode	3D (force2D = false)	2D shape features disabled
GLCM symmetry	Symmetrical GLCM enabled	—
Segment label	1	—
Interpolator	B-spline (sitkBSpline)	Relevant only when resampling is applied

Number of features per class

Class	Features
First-order	18
Shape (3D)	14
GLCM	24
GLRLM	16
GLSZM	16
GLDM	14
NGTDM	5
**Total**	**107**
