# Supplementary material for: Inter-Observer Reproducibility of [18F]FDG PET/CT Radiomic Features in Primary Breast Carcinoma
Source: J Imaging. 2026 Jul 4;12(7):300. doi: 10.3390/jimaging12070300 (PMC13413217; doi:10.3390/jimaging12070300)
Supplement: Supplementary file 1 [file jimaging-12-00300-s001.zip › Table S3.pdf]

Table S3. Inter-observer reproducibility across discretization and voxel-resampling configurations (sensitivity analysis).

All features were re-extracted and the inter-observer ICC analysis repeated for each configuration. High-stability features are those with ICC ≥ 0.90 and a 95% CI lower bound > 0.75. Agreement and rank-correlation are computed relative to the main configuration.

Configuration	Bin width (SUV)	Resampling	Median ICC (IQR)	Excellent (ICC ≥ 0.90)	High-stability	Reliability-class agreement vs main	Spearman ρ of per-feature ICCs vs main
Main study	0.25	None (native)	0.972 (0.905–0.991)	81	79	— (reference)	— (reference)
Sensitivity (finer)	0.125	None (native)	0.971 (0.922–0.991)	87	83	87.9%	0.966
Sensitivity (coarser)	0.5	None (native)	0.962 (0.888–0.990)	75	72	89.7%	0.982
Confirmatory (isotropic)	0.25	3 × 3 × 3 mm	0.976 (0.927–0.993)	83	80	88.8%	0.970

Reproducibility remained high across all configurations. The coarser bin width reduced the number of high-stability features from 79 to 72, consistent with the greater sensitivity of small-volume lesions to discretization, whereas isotropic resampling left the inter-observer ICC distribution essentially unchanged. Reliability-class agreement and Spearman ρ were calculated relative to the main configuration across all 107 features.
