# Supplementary figures and images for: Inter-Observer Reproducibility of [18F]FDG PET/CT Radiomic Features in Primary Breast Carcinoma
Source: J Imaging. 2026 Jul 4;12(7):300. doi: 10.3390/jimaging12070300 (PMC13413217; doi:10.3390/jimaging12070300)

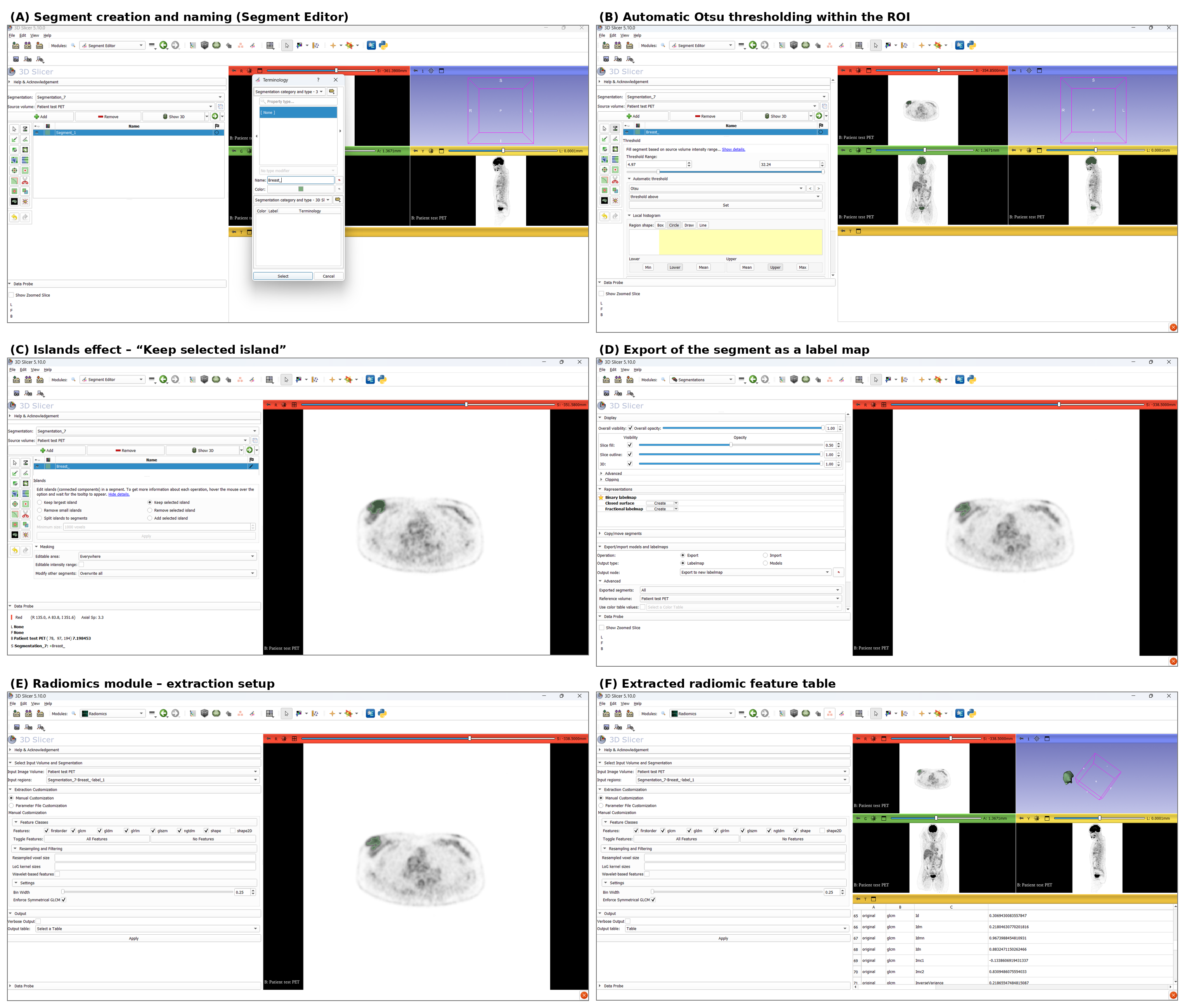

Supplement: Supplementary file 1 [file jimaging-12-00300-s001.zip › Figure S1.tiff]

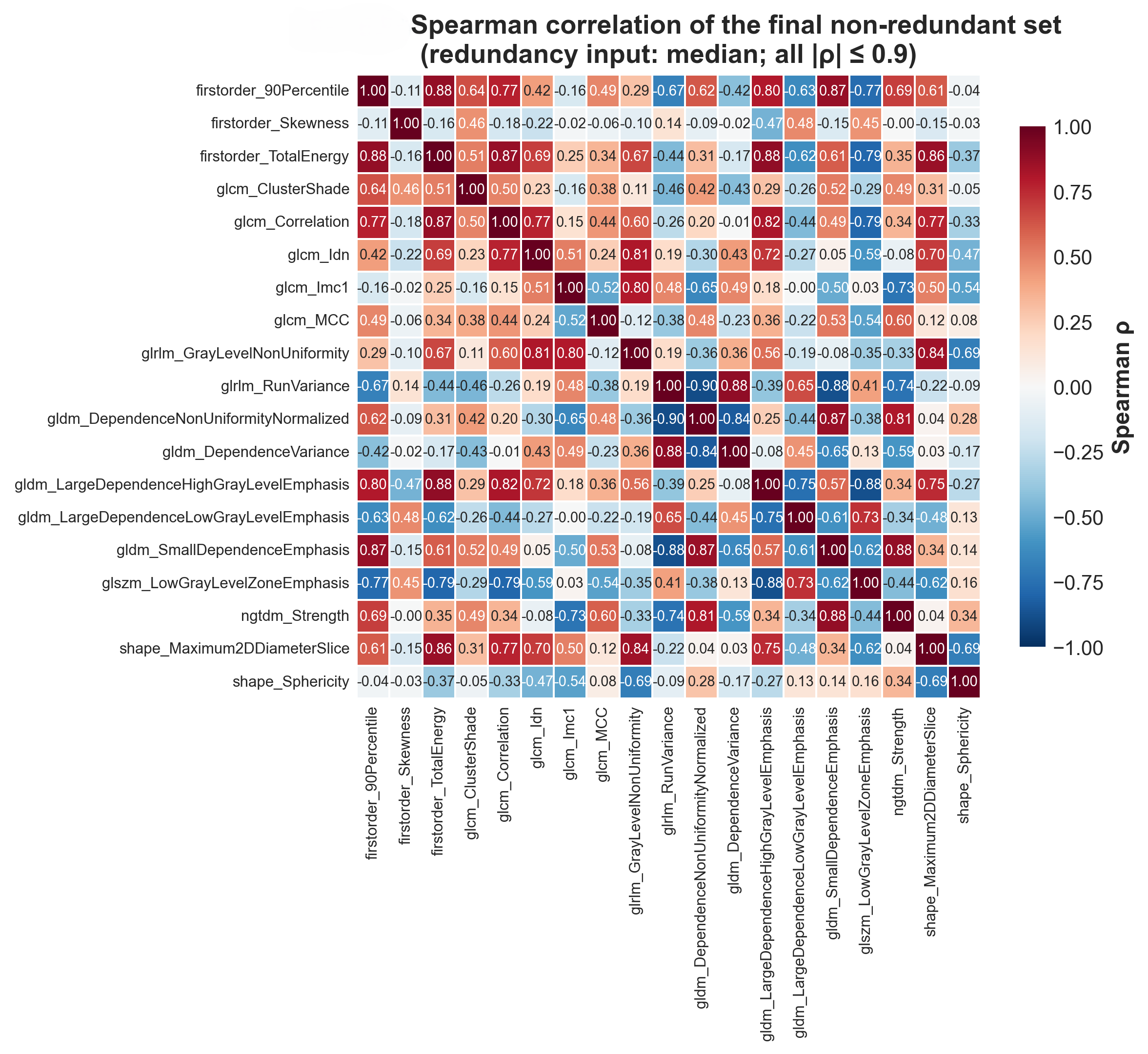

Supplement: Supplementary file 1 [file jimaging-12-00300-s001.zip › Figure S2.png]
